# Burden of Comorbid Conditions Among Individuals Screened for Lung Cancer

**DOI:** 10.1001/jamahealthforum.2024.5581

**Published:** 2025-02-21

**Authors:** Dejana Braithwaite, Shama Karanth, Christopher G. Slatore, Jae Jeong Yang, Martin Tammemagi, Michael K. Gould, Gerard A. Silvestri

**Affiliations:** 1 Division of Population Health Sciences, Department of Surgery, University of Florida College of Medicine, Gainesville; 2 University of Florida Health Cancer Canter, Gainesville; 3 Department of Epidemiology, University of Florida College of Medicine, Gainesville; 4 Center to Improve Veteran Involvement in Care, Portland VA Medical Center, Portland, Oregon; 5 Section of Pulmonary, Critical Care, Allergy, and Sleep Medicine, VA Portland Health Care System, Portland, Oregon; 6 Division of Pulmonary, Critical Care, and Allergy Medicine, Department of Medicine Oregon Health & Science University, Portland; 7 Department of Health Sciences, Brock University, St Catharines, Ontario, Canada; 8 Department of Health Systems Science, Kaiser Permanente Bernard J. Tyson School of Medicine, Pasadena, California; 9 Thoracic Oncology Research Group, Division of Pulmonary and Critical Care Medicine, Medical University of South Carolina, Charleston

## Abstract

**Importance:**

Screening for lung cancer with low-dose computed tomography (LDCT) has been shown to reduce lung cancer mortality in trials that included relatively younger, healthier, and predominantly White populations. The comorbidity profiles among patients undergoing lung cancer screening in practice settings are poorly understood.

**Objective:**

To evaluate the comorbidity profiles of patients in the Personalized Lung Cancer Screening (PLuS) cohort as a clinical setting vs the National Lung Screening Trial (NLST) participants in a clinical trial setting.

**Design, Setting, and Participants:**

This multicenter cohort study was conducted across 3 health care systems in California, Florida, and South Carolina and included patients who underwent LDCT lung cancer screening between 2016 and 2021. Data were analyzed between January 1, 2016, and December 31, 2021.

**Exposures:**

Receipt of the LDCT scan identified through *Current Procedural Terminology* and Healthcare Common Procedure Coding System codes.

**Main Outcomes and Measures:**

Detailed comorbidity data, measures of pulmonary function, and study data abstracted from electronic health records and institutional, Surveillance, Epidemiology, and End Results (SEER), and state registries were compared with self-reported comorbid conditions of participants in the LDCT arm of the NLST.

**Results:**

The PLuS cohort (n = 31 795) included 49.0% participants aged 65 years or older vs 26.6% in the NLST cohort (n = 26 723); 23.3% were individuals of racial and ethnic minority groups in the PLuS cohort compared with 8.5% in the NLST. The prevalence of comorbidity was substantially higher in the PLuS cohort than the NLST group, particularly chronic obstructive pulmonary disease (32.7% vs 17.5%), diabetes (24.6% vs 9.7%), and heart disease (15.9% vs 12.9%). Among those in the PLuS cohort, 19.3% had a Charlson Comorbidity Index score of 4 or higher, 18.0% had a frailty index greater than 0.20, 16.9% had a forced expiratory volume in 1 second (FEV-1) lower than 50% of predicted, and almost 5% had an ejection fraction lower than 40%. The prevalence of multimorbidity and frailty was especially high among those in the 75 years or older age group.

**Conclusions and Relevance:**

This study found that the PLuS cohort members were older, had greater illness severity, and more racially and ethnically diverse than the NLST participants. Older patients and those with consequential comorbidity likely had different risk-benefit profiles, which may have affected screening outcomes. The high prevalence of multimorbidity, frailty, and impaired cardiopulmonary function in the PLuS cohort suggests that the balance of benefits and harms observed in the NLST group may not translate to the clinical setting.

## Key Points

**Question:**

What are the comorbidity profiles of individuals undergoing lung cancer screening (LCS) with low-dose computed tomography in 3 integrated health systems?

**Findings:**

In this cohort study, the personalized lung cancer screening (PLuS) cohort (n = 31 795) of individuals undergoing LCS in California, Florida, and South Carolina was substantially more diverse in terms of age, race and ethnicity, and the comorbidity burden than National Lung Screening Trial (NLST) participants. The prevalence of comorbid conditions in the PLuS cohort was substantially higher than in the NLST, particularly chronic obstructive pulmonary disease, diabetes, and heart disease.

**Meaning:**

The balance of benefits and harms observed in the NLST may not translate to the clinical settings in which LCS takes place.

## Teaser

This cohort study compares the comorbidity profiles of patients undergoing lung cancer screening in practice settings vs clinical trials.

## Introduction

Lung cancer is the leading cause of cancer death in the US and worldwide,^1^ largely because most patients have advanced, incurable disease at the time of diagnosis. However, lung cancer screening (LCS) with low-dose computed tomography (LDCT) has the potential to revolutionize lung cancer outcomes through early detection. In 2011, the National Lung Screening Trial (NLST) demonstrated a 20% reduction in lung cancer mortality among current and former smokers randomly assigned to 3 rounds of annual LCS with LDCT compared with those assigned to 3 rounds of annual chest radiography.^2^ Given the number of participants experiencing a false-positive result over 3 years of annual screening, the rate of complications in procedures for benign nodules, the overall healthier status and younger age of NLST participants relative to screening-eligible adults in the clinical setting, and the higher procedure volumes and dedicated thoracic surgery support generally seen in NLST trial centers, it may prove difficult to replicate the relatively low risk of harms as LCS is implemented in clinical populations and settings.^3^ Compared with the NLST participants, individuals in the US eligible for LCS are nearly twice as likely to be aged 70 years or older and significantly more likely to currently smoke tobacco.^4^ Among the nearly 14 million LCS-eligible adults in the US, approximately 5 million have comorbid conditions that may diminish the overall benefit of screening.^5^

As part of the multicenter Personalized Lung Cancer Screening (PLuS) cohort study,^6^ we aimed to characterize the burden of comorbid disease in a patient population undergoing LCS in the clinical setting, especially when compared to the NLST cohort to provide insights into differences between the clinical LCS and trial populations.

## Methods

This retrospective cohort study of individuals who underwent LCS between January 1, 2016, and December 31, 2021, with no lung cancer diagnosis within 5 years preceding the LDCT, used data from electronic health records (EHR) and institutional, Surveillance, Epidemiology, and End Results (SEER), and State registries from 3 health care systems: the University of Florida (UF) Health, the Medical University of South Carolina (MUSC), and the Kaiser Permanente Southern California (KPSC). The study was approved by a single institutional review board at KPSC following ceding by the other 2 study sites, and consent was waived due to the use of deidentified data from electronic health records only.^6^ Sociodemographic variables (obtained from electronic health records and tumor registeries), including age, sex, race and ethnicity, height, weight, and smoking status, were obtained at the time of the index LDCT.^7^ Relevant clinical history, including the Charlson Comorbidity Score,^8^ the Elixhauser Comorbidity Score,^9^ the claims-based Frailty Index,^10^ results of spirometry and echocardiography, inpatient hospitalizations, and emergency department visits, was extracted from EHRs. Body mass index (BMI) was calculated from height and weight measurements and categorized according to the Centers for Disease Control guidelines. The participant characteristics in the LDCT arm of NLST were obtained from both the published literature^11^ and directly from the dataset. Statistical tests were conducted to compare the between-group differences on the study variables. Statistical analyses were conducted using SAS, version 9.4 (SAS Institute), and R, version 4.0 (R Foundation).

## Results

Compared with the LDCT arm of NLST (n = 26 723), PLuS participants (n = 31 795) were older, with nearly double the proportion of patients ages 70 to 74 years. The PLuS cohort included 49.0% participants aged 65 years or older vs 26.6% in the NLST; 3066 were 75 years or older in the PLuS cohort vs 1 patient in the NLST cohort (Table 1).

**Table 1.** Baseline Characteristics in PLuS and NLST Studies

Characteristic	No. (%)	
	PLuS (n = 31 795)	NLST (n = 26 723)
Age, y		
<55	1687 (5.3)	<2 (0.1)
55-59	6632 (20.9)	11 440 (42.8)
60-64	7894 (24.8)	8170 (30.6)
65-69	7523 (23.7)	4756 (17.8)
70-74	4993 (15.7)	2352 (8.8)
≥75	3066 (9.6)	1 (<0.1)
Sex		
Male	18 292 (57.5)	15 770 (59.0)
Female	13 503 (42.5)	10 953 (41.0)
Race and ethnicity		
American Indian or Alaska Native	152 (0.5)	92 (0.3)
Asian	2436 (7.7)	559 (2.1)
Black	4638 (14.6)	1196 (4.5)
Native Hawaiian or other Pacific Islander	179 (0.6)	91 (0.3)
White	21 751 (68.4)	24 289 (90.9)
More than 1 racial and ethnic group	NA	333 (1.3)
Unknown/missing	2639 (8.3)	163 (0.6)
Hispanic/Latino		
Hispanic/Latino	4337 (13.6)	479 (1.8)
Neither	24 405 (76.8)	26 080 (97.6)
Unknown/missing	3053 (9.6)	164 (0.6)
Smoking status		
Current	14 894 (46.8)	12 869 (48.2)
Former	13 607 (42.8)	13 854 (51.8)
Never	1758 (5.5)	
Missing	1536 (4.8)	
Smoking pack-years		
Median (IQR)	30 (15-42)	48 (39-66)
Missing	10 708 (33.7)	
BMI		
Underweight (<18.5)	789 (2.5)	245 (0.9)
Normal (18.5-24.9)	8522 (26.8)	7517 (28.1)
Overweight (25-29.9)	10 665 (33.5)	11 265 (42.2)
Obese (≥30.0)	10 423 (32.8)	7555 (28.3)
Missing	1396 (4.4)	141 (0.5)
Comorbidities^a^		
COPD/chronic bronchitis/emphysema	10 406 (32.7)	4674 (17.5)
Diabetes	7828 (24.6)	2594 (9.7)
Heart disease or myocardial infarction^b^	5057 (15.9)	3445 (12.9)
Stroke	743 (2.3)	753 (2.8)
Cancer^c^		
≥1	1702 (5.4)	1096 (4.1)

Abbreviations: BMI, body mass index (calculated as weight in kilograms divided by height in meters squared); COPD, chronic obstructive pulmonary disease; LDCT, low-dose computed tomography; NLST, national lung screening trial; PLuS, personalized lung cancer screening.

^a^ Comorbidities recorded within 1 year prior to baseline LDCT screening in the PLuS cohort.

^b^ Heart disease included congestive heart failure, cardiac arrythmias, myocardial infarction; data on individuals of more than 1 race or ethnic group were not available in the PLuS cohort.

^c^ Cancers recorded within 2 years prior to baseline LDCT in the PLuS cohort.

Table 2 provides a summary of the most common comorbidities/conditions, overall and by age group (eTable in Supplement 1). The prevalence of chronic obstructive pulmonary disease (COPD) in the PLuS cohort was almost double the prevalence in the NLST cohort (32.7% vs 17.5%), whereas the prevalence of diabetes in the PLuS cohort was more than double the prevalence in the NLST cohort (24.6% vs 9.7%). Among individuals in the PLuS cohort, 66.2% had at least 1 Charlson Comorbidity Index score and 19.3% scored 4 or higher on this index. Of those who underwent spirometry and/or echocardiography, 16.9% had forced expiratory volume in 1 second (FEV-1) lower than 50% of predicted and almost 5% had an ejection fraction lower than 40%. In-patient hospitalizations (25.2%) and emergency department visits (15.0%) were more common in the oldest age group (Table 2).

**Table 2.** Comorbid Conditions and Severity of Diseases in the PLuS Cohort^a^

Characteristic	No. (%)			
	Total (N = 31 795)	Age <65 y (n = 16 213)	Age 65 to ≤74 y (n = 12 516)	Age ≥75 y (n = 3066)
Charlson Comorbidity Index score				
0	10 729 (33.7)	7173 (44.2)	3207 (25.6)	349 (11.4)
1	8108 (25.5)	4278 (26.3)	3286 (26.3)	544 (17.7)
2	4351 (13.7)	1682 (10.3)	2088 (16.7)	581 (18.9)
3	2462 (7.7)	903 (5.6)	1145 (9.2)	414 (13.5)
≥4	6145 (19.3)	2177 (13.4)	2790 (22.3)	1178 (38.4)
Median (IQR)	1 (0-3)	1 (0-2)	1 (0-3)	3 (1-5)
Elixhauser Comorbidity score				
0	4738 (14.9)	3301 (20.4)	1315 (10.5)	122 (4.0)
1	5763 (18.1)	3544 (21.9)	1978 (15.8)	241 (7.9)
2	5593 (17.6)	2900 (17.9)	2245 (17.9)	448 (14.6)
3	4589 (14.4)	2067 (12.8)	2066 (16.5)	456 (14.9)
≥4	11 112 (34.9)	4401 (27.1)	4912 (39.3)	1799 (58.7)
Median (IQR)	2 (1-4)	2 (1-4)	3 (1-5)	4 (2-6)
Claims-Based Frailty Index score				
<0.05	6936 (21.8)	6763 (41.7)	173 (1.4)	0 (0.0)
0.05 to <0.10	9944 (31.3)	6818 (42.1)	3120 (24.9)	6 (0.2)
0.10 to <0.15	5728 (18.0)	1682 (10.4)	3897 (31.1)	149 (4.9)
0.15 to <0.20	3456 (10.9)	568 (3.5)	2455 (19.6)	433 (14.1)
≥ 0.20	5735 (18.0)	383 (2.4)	2873 (23.0)	2479 (80.8)
Median (IQR)	0.09 (0.05-0.17)	0.06 (0.04-0.08)	0.06 (0.04-0.08)	0.29 (0.22-0.40)
In-patient hospitalization (≥1)	6337 (19.9)	3344 (20.6)	2219 (17.7)	774 (25.2)
ED visits (≥1)	3088 (9.7)	1400 (8.6)	1229 (9.8)	459 (15.0)
FEV-1% predicted				
≥80%	1961 (42.2)	824 (46.9)	845 (38.5)	292 (42.0)
<80% to ≥50%	1899 (40.9)	678 (38.6)	937 (42.7)	284 (40.9)
<50% to ≥30%	675 (14.5)	209 (11.9)	359 (16.4)	107 (15.4)
<30%	113 (2.4)	47 (2.7)	54 (2.5)	12 (1.7)
Unknown	27 151 (85.4)	14 456 (89.1)	10 323 (82.5)	2372 (77.3)
Median (IQR)	75.3 (57.9-90.1)	78.2 (61.1-90.9)	73.2 (56.1-88.5)	75.3 (57.7-91.4)
Ejection fraction				
≥55%	4453 (82.7)	1615 (84.1)	2012 (82.4)	826 (81.0)
<55% to ≥40%	693 (12.9)	222 (11.2)	323 (13.2)	148 (14.5)
<40% to ≥30%	150 (2.8)	53 (2.5)	69 (2.8)	28 (2.8)
<30%	86 (1.6)	31 (1.3)	37 (1.5)	18 (1.8)
Unknown	26 417 (83.1)	14 293 (88.1)	10 077 (80.5)	2047 (66.8)
Median (IQR)	60.0 (55.0-64.7)	60.0 (57.0-63.6)	60.0 (55.0-65.0)	60.0 (55.0-62.0)

Abbreviations: ED, emergency department; FEV, forced expiratory volume; PLuS, personalized lung cancer screening.

^a^ Comorbid conditions were recorded within 1 year prior to baseline low-dose computed tomographic screening.

## Discussion

The current study found that patients undergoing LCS in the clinical setting were notably older, more racially and ethnically diverse, and had a higher burden of comorbidities, particularly among those aged 75 years and older, a group that was not included at baseline in the NLST. The prevalence of comorbidity was substantially higher in the PLuS than the NLST cohort, particularly COPD, diabetes and heart disease, and severe adverse comorbidity profiles, as reflected by the Charlson Comorbidity Index score of 4 or higher (19.3%) and severe lung obstruction based on pulmonary function tests (16.9%). Our study population included individuals screened for lung cancer with LDCT, including some never smokers for whom the benefits of screening are unlikely to outweigh the harms. The underlying comorbid conditions, heavy smoking history, and aging of this population may adversely affect health outcomes throughout the screening continuum from diagnostic testing for screen-detected abnormalities, to the ability to undergo potentially curative surgery for lung cancer.^12^ In addition, competing causes of death from diseases such as COPD, heart disease, and other cancers may attenuate the benefit of screening.^12^ Our findings point to the greater racial and ethnic diversity in the LCS population as a meaningful step toward reducing disparities in outcomes. Observing a higher proportion of individuals of racial and ethnic minority groups in practice settings vs in the NLST trial provides an opportunity to better understand how LCS performs in these subgroups who have historically had higher rates of comorbid conditions, worse adherence to follow-up LCS, worse outcomes from cancer treatment, and decreased access to health care.^13^ There is an opportunity to develop and deploy new interventions that manage the complex health profiles of older LCS candidates with greater disease severity and target LCS in populations where it is likely to have the greatest impact.^14^

### Strengths and Limitations

This cohort study had design features that overcame limitations of some earlier studies. It included a relatively large LDCT screening cohort from 3 integrated and geographically diverse health care systems, capturing granular data on comorbidities and representing populations outside of a clinical trial setting, making the results more generalizable to those undergoing LDCT screening in the US. Comorbidity data were collected systematically in detail from medical records, which are generally considered superior to data collected from administrative databases. We have incorporated measures of pulmonary function and novel indicators of frailty—factors that more precisely capture the severity of comorbid conditions and build on existing studies,^13^ which have focused exclusively on comorbidity type and number. This represents a major innovation and step toward precision LCS. Limitations include the inability to capture relevant exposures such as vaping from EHRs, which may influence lung cancer risk.

## Conclusions

In this cohort study, patients in the PLuS cohort undergoing screening for lung cancer outside of clinical trials were older, had a higher comorbidity burden, and were more racially and ethnically diverse than participants in the NLST clinical trial. These findings suggest that many individuals screened for lung cancer may experience additional complications in conjunction with lung cancer diagnosis and treatment because of their age and underlying comorbid conditions and may not live long enough to benefit from LCS. Older patients and those with consequential comorbidity likely have different risk-benefit profiles, which may affect screening outcomes. Our results underscore the importance of developing the evidence for advancing risk-based LCS.

## Notes

Supplement 1. eTable. ICD 9 and 10 Codes for Included Comorbidities

Supplement 2. Data Sharing Statement
