# Supplementary material for: Burden of Comorbid Conditions Among Individuals Screened for Lung Cancer
Source: JAMA Health Forum. 2025 Feb 21;6(2):e245581. doi: 10.1001/jamahealthforum.2024.5581 (PMC11846005; doi:10.1001/jamahealthforum.2024.5581)
Supplement: Supplement 1. — eTable. ICD 9 and 10 Codes for Included Comorbidities [file jamahealthforum-e245581-s001.pdf]

Supplemental Online Content

Braithwaite D, Karanth S, Slatore CG, et al. Burden of comorbid conditions among individuals screened for lung cancer in 3 integrated health systems. *JAMA Health Forum*. 2025;6(2):e245581. doi:10.1001/jamahealthforum.2024.5581

eTable. ICD 9 and 10 Codes for Included Comorbidities

This supplemental material has been provided by the authors to give readers additional information about their work.

© 2025 Braithwaite D, et al. *JAMA Health Forum*.

eTable. ICD 9 and 10 Codes for Included Comorbidities

**Part A. Charleston Comorbidity Index**		
**Comorbidity**	**ICD-9**	**ICD-10**
Myocardial infarction	I21, I22, I25.2	410, 412
Congestive heart failure	I09.9, I11.0, I13.0, I13.2, I25.5, I42.0, I42.5–I42.9, I43, I50, P29.0	398.91, 402.01, 402.11, 402.91, 404.01, 404.03, 404.11, 404.13, 404.91, 404.93, 425.4–425.9, 428
Cerebrovascular disease	G45, G46, H34.0, I60–I69	362.34, 430–438
Dementia	F00–F03, F05.1, G30, G31.1	290, 294.1, 331.2
Rheumatologic disease	M05, M06, M31.5, M32–M34, M35.1, M35.3, M36.0	446.5, 710.0–710.4, 714.0– 714.2, 714.8, 725
Peptic ulcer disease	K25–K28	531–534
Liver disease, *mild*	B18, K70.0–K70.3, K70.9, K71.3–K71.5, K71.7, K73, K74, K76.0, K76.2–K76.4, K76.8, K76.9, Z94.4	070.22, 070.23, 070.32, 070.33, 070.44, 070.54, 070.6, 070.9, 570, 571, 573.3, 573.4, 573.8, 573.9, V42.7
Liver disease, *moderate-severe*	I85.0, I85.9, I86.4, I98.2, K70.4, K71.1, K72.1, K72.9, K76.5, K76.6, K76.7	456.0–456.2, 572.2–572.8
Diabetes, *uncomplicated*	E10.0, E10.1, E10.6, E10.8, E10.9, E11.0, E11.1, E11.6, E11.8, E11.9, E12.0, E12.1, E12.6, E12.8, E12.9, E13.0, E13.1, E13.6, E13.8, E13.9, E14.0, E14.1, E14.6, E14.8, E14.9	250.0–250.3, 250.8, 250.9
Diabetes, *complicated*	E10.2–E10.5, E10.7, E11.2–E11.5, E11.7, E12.2–E12.5, E12.7, E13.2–E13.5, E13.7, E14.2–E14.5, E14.7	250.4–250.7
Renal disease	I12.0, I13.1, N03.2–N03.7, N05.2–N05.7, N18, N19, N25.0, Z49.0–Z49.2, Z94.0, Z99.2	403.01, 403.11, 403.91, 404.02, 404.03, 404.12, 404.13, 404.92, 404.93, 582, 583.0–583.7, 585, 586, 588.0, V42.0, V45.1, V56
Malignancy, *including leukemia or lymphoma*	C00–C26, C30–C34, C37– C41, C43, C45–C58, C60– C76, C81–C85, C88, C90–C97	140–172, 174–195.8, 200–208, 238.6
**Part B. Elixhauser Comorbidity Index**		
**Comorbidity**	**ICD-9**	**ICD-10**
Congestive heart failure	I09.9, I11.0, I13.0, I13.2, I25.5, I42.0, I42.5– I42.9, I43, I50, P29.0	398.91, 402.01, 402.11, 402.91, 404.01, 404.03, 404.11, 404.13, 404.91, 404.93, 425.5, 425.6, 425.7, 425.8, 425.9, 428.0, 428.1, 428.20, 428.21, 428.22, 428.23, 428.30, 428.31, 428.32, 428.33, 428.40, 428.41, 428.42, 428.43, 428.9
Cardiac arrhythmias	I44.1-144.3, 145.6, 145.9, I47-I49, R00.0, R00.1, R00.8, T82.1, Z45.0, Z95.0<br>F102	426.0, 426.10, 426.12, 426.13, 426.7, 426.9, 427.0-427.4, 427.6-427.9, 785.0,996.01, 996.04, V45.0, V53.3
Valvular disease	A52.0, I05-I08, I09.1, I09.8, I34-I39, Q23.0-Q23.3, Z95.2-Z95.4	093.2, 394-397, 424, 746.3-746.6, V42.2,V43.3
Pulmonary circulation disorders	I26-I27, I28.0, I28.8, I28.9<br>F108	415.0, 415.1, 416, 417.0, 417.8, 417.9

© 2025 Braithwaite D, et al. *JAMA Health Forum*.

Peripheral vascular disorders	I70, I71, I73.1, I73.8, I73.9, I77.1, I79.0, I79.2, K55.1, K55.8, K55.9, Z95.8, Z95.9	093.0, 437.3, 440,441, 443.1-443.9, 447.1, 557.1, 557.9, V43.4
Hypertension, *uncomplicated*	I10	401
Hypertension, *complicated*	I11, I12, I13, I15	402-405
Paralysis	G04.1, G11.4, G80.1, G80.2, G81-G82, G83.0-G83.4, G83.9	334.1, 342, 343, 344.0-344.6, 344.9
Other neurological disorders	G10–G13, G20– G22, G25.4, G25.5, G31.2, G31.8, G31.9, G32, G35–G37, G40, G41, G93.1, G93.4, R47.0, R56	331.9, 332.0, 332.1, 333.4, 333.5, 333.92, 334–335, 336.2, 340, 341, 345, 348.1, 348.3, 780.3, 784.3
Chronic pulmonary disease	I27.8, 127.9, J40-J47, J60-J67, J68.4, J70.1, J70.3	416.8, 416.9, 490-505, 506.4, 508.1, 508.8
Diabetes, uncomplicated	E10.0, E10.1, E10.9, E11.0, E11.1, E11.9, E12.0, E12.1, E12.9, E13.0, E13.1, E13.9, E14.0, E14.1, E14.9	250.0-250.3
Diabetes, complicated	E10.2-E10.8, E11.2-E11.8, E12.2-E12.8, E13.2-E13.8, E14.2-E14.8	250.4-250.9
Hypothyroidism	E00-E03, E89.0<br>F139	240.9, 243, 244, 246.1, 246.8
Renal failure	I12.0, I13.1, N18-N19, N25.0, Z49.0– Z49.2, Z94.0, Z99.2	403.01, 403.11, 403.91, 404.02, 404.03, 404.12, 404.13, 404.92, 404.93, 585-586, 588.0, V42.0, V45.1, V56
Liver disease	B18, I85, I86.4, I98.2, K70, K71.1, K71.3-K71.5, K71.7, K72-K74, K76.0, K76.2-K76.9, Z94.4	070.22, 070.23, 070.32, 070.33, 070.44, 070.54, 070.6, 070.9, 456.0-456.2, 570-571, 572.2-572.8, 573.3, 573.4, 573.8, 573.9, V42.7
Peptic ulcer disease	K25.7, K25.9, K26.7, K26.9, K27.7, K27.9, K28.7, K28.9	531.7, 531.9, 532.7, 532.9, 533.7, 533.9, 534.7, 534.9
AIDS	B20-B22, B24	042-044
Lymphoma	C81-C85, C88, C96, C90.0, C90.2	200-202, 203.0, 238.6
Metastatic cancer	C77, C78, C79, C80	196-199
Solid tumor, *no metastasis*	C00-C26, C30-C34, C37-C41, C43, C45-C58, C60-C76, C97	140-172, 174-195
Rheumatoid arthritis	L94.0, L94.1, L94.3, M05-M06, M08, M12.0, M12.3, M30, M31.0-M31.3, M32-M35, M45, M46.1, M46.8, M46.9	446, 701.0, 710.0– 710.4, 710.8, 710.9, 711.2, 714, 719.3, 720, 725, 728.5, 728.89, 729.30
Coagulopathy	D65-D68, D69.1, D69.3-D69.6	286, 287.1, 287.3-287.5
Obesity	E66	278.0
Weight Loss	E40-E46, R63.4, R64	260-263, 783.2, 799.4
Fluid & Electrolyte disorders	E22.2, E86, E87	253.6, 276
Blood loss anemia	E185	D50.0
Deficiency anemia	D50.8, D50.9, D51-D53	280.1-280.9, 281
Alcohol abuse	F10, E52, G62.1, I42.6, K29.2, K70.0, K70.3, K70.9, T51, Z50.2, Z71.4, Z72.2	265.2, 291.1-291.3, 291.5-291.9, 303.0,303.9, 305.0, 357.5, 425.5, 535.3, 571.0-571.3, 980, V11.3
Drug abuse	F11-F16, F18, F19, Z71.5, Z72.2	292, 304, 305.2-305.9, V65.42
Psychoses	F20, F22-F25, F28, F29, F30.2, F31.2, F31.5	293.8, 295, 296.04, 296.14, 296.44, 296.54, 297, 298
Depression	F20.4, F31.3-F31.5, F32-F33, F34.1, F41.2, F43.2	296.2, 296.3, 296.5, 300.4, 309, 311

© 2025 Braithwaite D, et al. *JAMA Health Forum*.

Part C. Claims-Based Frailty Index		
**Comorbidity**	**ICD-9**	**ICD-10**
Impaired mobility	Z74.0-Z74.9	V46.3
Depression	F06.30, F30.10, F30.11, F30.12, F30.13, F30.2, F30.3, F30.4, F30.8, F31.10, F31.11, F31.12, F31.13, F31.2, F31.30, F31.31, F31.32, F31.4, F31.5, F31.60, F31.61, F31.62, F31.63, F31.64, F31.73, F31.74, F31.75, F31.76, F31.77, F31.78, F31.81, F31.9, F32.0, F32.1, F32.2, F32.3, F32.4, F32.5, F32.89, F32.9, F32.A, F33.0, F33.1, F33.2, F33.3, F33.40, F33.41, F33.42, F33.8 F33.9, F34.1, F34.81, F34.89, F43.20, F43.21, F43.22, F43.23, F43.24, F43.25, F43.29, F43.8, F94.8<br>F252	309.0, 309.1, 309.22, 309.23, 309.24, 309.28, 309.29, 309.3, 309.4, 309.82, 309.83, 309.89, 309.9, 293.83, 296.00, 296.01, 296.02, 296.03, 296.04, 296.05, 296.06, 296.10, 296.11, 296.12, 296.13, 296.14, 296.15, 296.16, 296.20, 296.21, 296.22, 296.23, 296.24, 296.25, 296.26, 296.30, 296.31, 296.32, 296.33, 296.34, 296.35, 296.36, 296.40, 296.41, 296.42, 296.43, 296.44, 296.45, 296.46, 296.50, 296.51, 296.52, 296.53, 296.54, 296.55, 296.56, 296.60, 296.61, 296.62, 296.63, 296.64, 296.65, 296.66, 296.7, 296.80, 296.81, 296.82, 296.89, 296.90, 296.99, 300.4, 311
Congestive Heart Failure	I09.81, I50.0 - I50.41, I50.43-I50.9	398.91, 428.0, 428.1, 428.20, 428.21, 428.22, 428.23, 428.30, 428.31, 428.32, 428.33, 428.40, 428.41, 428.43, 428.9
Parkinson’s disease	G20, G21	332
Arthritis	M05, M06.1, M06.4, M06.9, M08, M12, M15.0, M15.1, M15.2, M15.3, M15.8, M15.9, M16, M17, M18.9, M19, M45.9	714.0, 714.1, 714.2, 714.30, 714.31, 714.32, 714.33, 714.4, 714.81, 714.89, 714.9, 715.00, 715.04, 715.09, 715.10, 715.11, 715.12, 715.13, 715.14, 715.15, 715.16, 715.17, 715.18, 715.20, 715.21, 715.22, 715.23, 715.24, 715.25, 715.26, 715.27, 715.28, 715.30, 715.31, 715.32, 715.33, 715.34, 715.35, 715.36, 715.37, 715.38, 715.80, 715.89, 715.90, 715.91, 715.92, 715.93, 715.94, 715.95, 715.96, 715.97, 715.98, 720.0, V13.4
Cognitive impairment	F01.50, F01.51, F02.80, F02.81, F03.90, F03.91, F04, F05, F06.0, F06.8, F07.0, F07.81, F07.89, F09, F48.2, G30.9, G31.01, G31.09, G31.1, G31.83	290.0, 290.10, 290.11, 290.12, 290.13, 290.20, 290.21, 290.3, 290.40, 290.41, 290.42, 290.43, 290.8, 290.9, 293.0, 293.1, 294.0, 294.1, 294.10, 294.11, 294.20, 294.21, 294.8, 294.9, 310.0, 310.2, 310.8, 310.81, 310.89, 310.9, 331.0, 331.1, 331.11, 331.19, 331.2, 331.82, 797
Stroke	G43.6, G45.0, G45.1, G45.2, G45.3, G45.8, G45.9, G46.0, G46.1, G46.2, G46.3, G46.4, G46.5, G46.6, G46.7, G46.8, G97.31, G97.32, I60.00, I60.01, I60.02, I60.10, I60.11, I60.12, I60.2, I60.20, I60.21, I60.22, I60.30,	346.60, 346.61, 346.62, 346.63, 430, 431, 432.0, 432.1, 432.9, 433.01, 433.11, 433.21, 433.31, 433.81, 433.91, 434.0, 434.00, 434.01, 4341, 434.10, 434.11, 434.9, 434.90, 434.91, 436, 438.13, 438.14, 438.19, 438.20, 438.21, 438.22, 438.40,

© 2025 Braithwaite D, et al. *JAMA Health Forum*.

	I60.31, I60.32, I60.4, I60.50, I60.51, I60.52, I60.6, I60.7, I60.8, I60.9, I61.0, I61.1, I61.2, I61.3, I61.4, I61.5, I61.6, I61.8, I61.9, I62.00, I62.01, I62.02, I62.9, I63.00, I63.011, I63.012, I63.013, I63.019, I63.02, I63.031, I63.032, I63.033, I63.039, I63.09, I63.10, I63.111, I63.112, I63.113, I63.119, I63.12, I63.131, I63.132, I63.133, I63.139, I63.19, I63.20, I63.211, I63.212, I63.213, I63.219, I63.22, I63.231, I63.232, I63.233, I63.239, I63.29, I63.30, I63.311, I63.312, I63.313, I63.319, I63.321, I63.322, I63.323, I63.329, I63.331, I63.332, I63.333, I63.339, I63.341, I63.342, I63.343, I63.349, I63.39, I63.40, I63.411, I63.412, I63.413, I63.419, I63.421, I63.422, I63.423, I63.429, I63.431, I63.432, I63.433, I63.439, I63.441, I63.442, I63.443, I63.449, I63.49, I63.50, I63.511, I63.512, I63.513, I63.519, I63.521, I63.522, I63.523, I63.529, I63.531, I63.532, I63.533, I63.539, I63.541, I63.542, I63.543, I63.549, I63.59, I63.6, I63.8, I63.81, I63.89, I63.9, I67.841, I67.848, I67.89, I97.810, I97.811, I97.820, I97.821	438.41, 438.42, 438.50, 438.51, 438.52, 438.53, 438.6, 438.7, 438.81, 438.82, 438.83, 438.84, 438.85, 438.89, 438.9
Paranoia	F06.0, F06.2, F20-F29	293.81, 293.82, 295.00, 295.01, 295.02, 295.03, 295.04, 295.05, 295.10, 295.11,295.12, 295.13, 295.14, 295.15, 295.20, 295.21, 295.22, 295.23, 295.24, 295.25, 295.30, 295.31, 295.32, 295.33, 295.34, 295.35, 295.40, 295.41, 295.42, 295.43, 295.44, 295.45, 295.50, 295.51, 295.52, 295.53, 295.54, 295.55, 295.60, 295.61, 295.62, 295.63, 295.64, 295.65, 295.70, 295.71, 295.72, 295.73, 295.74,295.75, 295.80, 295.81, 295.82, 295.83, 295.84, 295.85, 295.90, 295.91, 295.92, 295.93, 295.94, 295.95, 297.0, 297.1, 297.2, 297.3, 297.8, 297.9, 298.0, 298.1,298.2, 298.3, 298.4,298.8, 298.9
Chronic skin ulcers	L89, L97, L98.4	707.0, 707.00, 707.01, 707.02, 707.03, 707.04, 707.05, 707.06, 707.07, 707.09, 707.1, 707.10, 707.11, 707.12, 707.13, 707.14, 707.15, 707.19, 707.20, 707.21, 707.22, 707.23, 707.24, 707.25, 707.8, 707.9

© 2025 Braithwaite D, et al. *JAMA Health Forum*.

Pneumonia	A01.03, A02.22, A20.2, A21.2, A22.1, A31.0, A37.91, A40.3, A42.0, A43.0, A48.1, A50.04, A54.84, A78, B01.2, B05.2, B25.0, B37.1, B38.0, B38.1, B38.2, B39.0, B39.2, B39.9, B40.0, B40.2, B44.0, B58.3, B59, B95.3, J10.00, J10.01, J10.08, J12.0, J12.1, J12.2, J12.3, J12.81, J12.82, J12.89, J12.9, J13, J14, J15.0, J15.1, J15.20, J15.211, J15.212, J15.29, J15.3, J15.4, J15.5, J15.6, J15.7, J15.8, J15.9, J16.0, J16.8, J17, J18.0, J18.1, J18.2, J18.8, J18.9, J20.0, J85.1, P23.0, P23.1, P23.2, P23.3, P23.4, P23.5, P23.6, P23.8	003.22, 020.3, 020.4, 020.5, 021.2, 022.1, 031.0, 039.1, 052.1, 055.1, 073.0,083.0, 112.4, 114.0, 114.4, 114.5, 115.05, 115.15, 115.95, 130.4, 136.3, 480.0, 480.1, 480.2, 480.3, 480.8, 480.9, 481, 482.0, 482.1, 482.2, 482.3, 482.30, 482.31, 482.32, 482.39, 482.4, 482.40, 482.41, 482.42, 482.49, 482.8, 482.81, 482.82, 482.83, 482.84, 482.89, 482.9, 483, 483.0, 483.1, 483.8, 484.1, 484.3, 484.5, 484.6, 484.7, 484.8, 485, 486, 513.0, 517.1
Skin & soft tissue infection	A20.1, A21.0, A22.0, A31.1, A36.3, A46, L00-L08	020.1, 021.0, 022.0, 031.1, 032.85, 035., 039.0, 680.0, 680.1, 680.2, 680.3, 680.4, 680.5, 680.6, 680.7, 680.8, 680.9, 681.00, 681.01, 681.02, 681.10, 681.11, 681.9, 682.0, 682.1, 682.2, 682.3, 682.4, 682.5, 682.6, 682.7, 682.8, 682.9, 684., 685.0, 685.1, 686.0, 686.00, 686.01, 686.09, 686.1, 686.8, 686.9
Mycoses	B35-B49	110.0, 110.1, 110.2, 110.3, 110.4, 110.5, 110.6, 110.8, 110.9, 111.0, 111.1, 111.2, 111.3, 111.8, 111.9, 112.0, 112.1, 112.2, 112.3, 112.5, 112.82, 112.84, 112.85, 112.89, 112.9, 114.1, 114.3, 114.9, 115.00, 115.09, 115.10, 115.19, 115.90, 115.99, 116.0, 116.1, 116.2, 117.0, 117.1, 117.2, 117.3, 117.4, 117.5, 117.6, 117.7, 117.8, 117.9, 118
Gout or other crystal-induced arthropathy	M10, M11	274.0, 274.00, 274.01, 274.02, 274.03, 274.10, 274.11, 274.19, 274.81, 274.82, 274.89, 274.9, 712.10, 712.11, 712.12, 712.13, 712.14, 712.15, 712.16, 712.17, 712.18, 712.19, 712.20, 712.21, 712.22, 712.23, 712.24, 712.25, 712.26, 712.27, 712.28, 712.29, 712.30, 712.31, 712.32, 712.33, 712.34, 712.35, 712.36, 712.37, 712.38, 712.39, 712.80, 712.81, 712.82, 712.83, 712.84, 712.85, 712.86, 712.87, 712.88, 712.89, 712.90, 712.91, 712.92, 712.93, 712.94, 712.95, 712.96, 712.97, 712.98, 712.99
Falls	W00-W19	E8800, E8801, E8809, E8810, E8811, E882, E8830, E8831, E8832, E8839, E8840, E8841, E8842, E8843, E8844, E8845, E8846, E8849, E885, E8850, E8851, E8852,

© 2025 Braithwaite D, et al. *JAMA Health Forum*.

		E8853, E8854, E8859, E8860, E8869, E888, E8880, E8881, E8888, E8889, E9681, E9870, E9871, E9872, E9879
Musculoskeletal problems	M00-M99	713.X, 716.XX, 718.XX, 719.XX, 720.X, 721.XX, 722.XX, 723.X, 724.X, 733.XX, V13.51, 435.X
Urinary tract infection	A36.85, N10, N11.0, N11.8, N12, N15.1, N15.9, N16, N28.84, N28.85, N28.86, N30.00, N30.01, N30.10, N30.11, N30.20, N30.21, N30.30, N30.31, N30.40, N30.41, N30.80, N30.81, N30.90, N30.91, N34.0, N34.1, N34.2, N34.3, N35.111, N37	032.84, 590.00, 590.01, 590.10, 590.11, 590.2, 590.3, 590.80, 590.81, 590.9, 595.0, 595.1, 595.2, 595.3, 595.4, 595.81, 595.82, 595.89, 595.9, 597.0, 597.80, 597.81, 597.89, 598.00, 598.01, 599.0

© 2025 Braithwaite D, et al. *JAMA Health Forum*.
