# Supplementary material for: Burden of Comorbid Conditions Among Individuals Screened for Lung Cancer
Source: JAMA Health Forum. 2025 Feb 21;6(2):e245581. doi: 10.1001/jamahealthforum.2024.5581 (PMC11846005; doi:10.1001/jamahealthforum.2024.5581)
Supplement: Supplement 2. — Data Sharing Statement [file jamahealthforum-e245581-s002.pdf]

Data Sharing Statement

Braithwaite. Burden of Comorbid Conditions Among Individuals Screened for Lung Cancer. *JAMA Health Forum*. Published February 21, 2025. doi:10.1001/jamahealthforum.2024.5581

Data
Data available: No

Additional Information
Explanation for why data not available: We will make the data available to others once the project is complete and upon request.
